# Supplementary material for: Phenotypic Heterogeneity of Variably Protease-Sensitive Prionopathy: A Report of Three Cases Carrying Different Genotypes at PRNP Codon 129
Source: Viruses. 2022 Feb 10;14(2):367. doi: 10.3390/v14020367 (PMC8879235; doi:10.3390/v14020367)
Supplement: Supplementary file 1 [file viruses-14-00367-s001.zip › viruses-1520307-supplementary.pdf]

viruses

Supplementary Materials

Phenotypic Heterogeneity of Variably Protease-Sensitive Prionopathy: A Report of Three Cases Carrying Different Genotypes at *PRNP* Codon 129

Simone Baiardi [1,2], Angela Mammana [1,2], Marcello Rossi [1], Anna Ladogana [3], Benedetta Carlà [1], Pierluigi Gambetti [4], Sabina Capellari [1,5] and Piero Parchi [1,2,*]

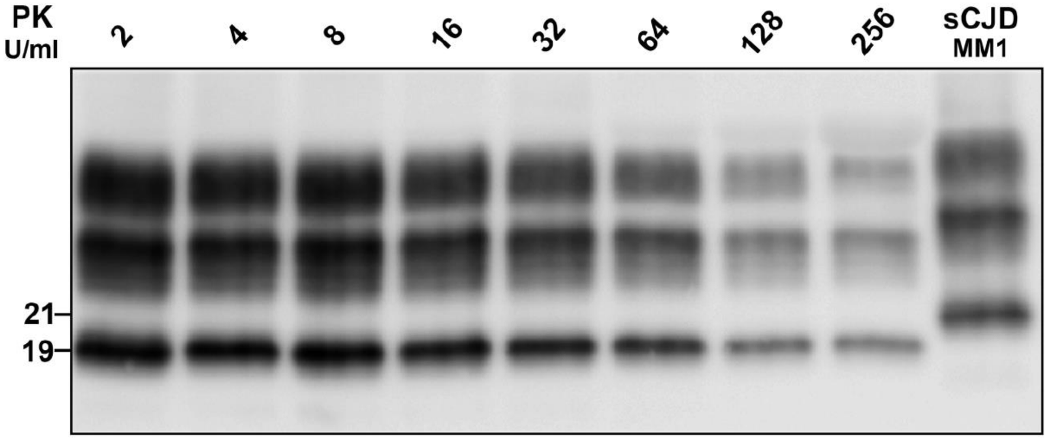

Figure S1. Analysis of protease resistance of PrP^{Sc} by PK titration assay in a sCJD VV2 brain. A frontal cortex brain homogenate was run after digestion with increasing amounts of PK (range from 2 to 256 U/mL). A progressive reduction of PrP^{Sc} signal intensity was identified for PC concentration >32 U/mL, whereas in the most PK resistant VPSPr case (129MM) a remarkable loss of signal was already detected using PK at 4 U/mL. sCJD MM1 was included as a control, Membranes were probed with mAb 3F4. Approximate molecular masses are in kilodaltons.
